# Molecular Interactions Between Dimethylated Arginine and the Nitric Oxide Axis Unveil Programmed Death-Ligand 1 (PD-L1) Signaling Signatures in Gastric Cancer

**DOI:** 10.7759/cureus.103478

**Published:** 2026-02-12

**Authors:** Priyatma, Shyam Prakash, Govind K Makharia, Siddhartha D Gupta, Peush Sahni, Ranjit K Sahoo, Sanjay Thulkar, Arulselvi Subramanian, R. M Pandey

**Affiliations:** 1 Laboratory Medicine, All India Institute of Medical Sciences, New Delhi, New Delhi, IND; 2 Gastroenterology, All India Institute of Medical Sciences, New Delhi, New Delhi, IND; 3 Pathology, All India Institute of Medical Sciences, New Delhi, New Delhi, IND; 4 Gastrointestinal Surgery, All India Institute of Medical Sciences, New Delhi, New Delhi, IND; 5 Medical Oncology, All India Institute of Medical Sciences, New Delhi, New Delhi, IND; 6 Radiodiagnosis and Interventional Radiology, All India Institute of Medical Sciences, New Delhi, New Delhi, IND; 7 Laboratory Medicine, Jay Prakash Narayan Trauma Centre, All India Institute of Medical Sciences, New Delhi, New Delhi, IND; 8 Biostatistics, All India Institute of Medical Sciences, New Delhi, New Delhi, IND

**Keywords:** dimethylation, enos, gastric cancer, mrna, pd-l1, tight junction proteins

## Abstract

Background

Gastric cancer (GC) is one of the most common cancers globally. Programmed death cells, a cell-surface molecule, drive arginine dimethylation, disrupting nitric oxide (NO) production in peripheral tissues. Programmed cell death protein 1 (PD-1)/programmed death-ligand 1 (PD-L1) axis disruption depends on dimethylarginine dimethylaminohydrolase 1 (DDAH1) activity during metastasis in patients with severe gastritis, either synergizing with NO or inhibiting PD-1/PD-L1 activation in tumor growth. This study aimed to determine the arginine dimethylation process in conjunction with nitrosative stress, which dysregulates the PD-L1 axis in GC cells.

Methodology

A cross-sectional study was conducted utilizing real-time polymerase chain reaction for relative mRNA expressions, high-performance liquid chromatography for asymmetric dimethylarginine (ADMA)/symmetric dimethylarginine (SDMA) assays, and spectrophotometry for NO analysis. Statistical tools such as RStudio (version 2024.12.1) were used to conduct principal component analysis, heatmaps, and t-tests/analysis of variance or Mann-Whitney/Kruskal-Wallis tests after Shapiro-Wilk post-hoc tests in different groups (GC, disease control, and healthy control).

Results

We observed abnormal NO production and reduced mitochondrial DNA copy numbers in GC patients. Significantly decreased levels of ADMA and excessive influx of arginase activity were determined in GC patients. PD-L1 expression was significantly higher in GC patients, while suboptimal PD-L1 expression was associated with disease control. The abnormal influx of dimethylated arginine and matrix metalloproteinase-7 (MMP-7) was associated with and linked to NO production levels. Their association could be with the nitrigenic pathway and with possible mechanisms for damaging cell-surface molecules in GC.

Conclusions

Overall, the disrupted ADMA-SDMA balance and Ca++ permeability impair the regulation of claudin-4, MMP-7, and PD1/PD-L1 in GC patients. These variables hold promise as diagnostic and therapeutic targets for improved GC management.

## Introduction

Gastric cancer (GC) is the fourth most common oncological abnormality after breast, skin, and lung cancer [1]. It can develop in any part of the stomach and spread to other organs, especially in the lungs, liver, bones, and esophagus (the cardia). Programmed cell death protein 1 (PD-1) and its ligand, i.e., programmed death-ligand 1 (PD-L1) (PD-L1)/programmed death-ligand 2 (PD-L2), are normally involved as a negative regulator in various tumors. Thus, a disruption of the PD-1 and PD-L1 axis in severe gastritis is firmly associated with invasion and lymphatic metastasis in cancer patients. However, they are poorly defined in stomach-oriented tumors. PD-L1 mRNA is expressed in various cancer cells and actively induces the evasion of host T cells. Blockade of PD-1/PD-L1 in cancer cells has been extensively studied in anti-tumor immunity and tumor growth inhibition in cancer patients [2]. The regulation of PD-L1 expression remains largely unknown in GC. However, some studies have been reported on advanced-stage cancer, at the junctions of adenocarcinoma with immune system evasion, and on the checkpoints of immune-expressed molecules [3]. The trials of metabolic programming are recognized as a hallmark of cancer and progression due to the Warburg effect [4].

Although the targeted and non-targeted metabolic profiles at advanced-stage arginine are the key substrates involved repeatedly and dysregulated at varying rates across the types of cancer, they remain constant over the period of cancer. Either they reflect in a heterogeneous manner or progress toward the end stage of cancer [5]. Nitrigenic enzymes have been frequently associated with reactive nitrogen species and dysregulate the nitric oxide (NO) behavior depending on asymmetric dimethylarginine (ADMA) or symmetric dimethylarginine (SDMA) concentration in various cancers [6,7]. However, the nitrigenic pathway is not yet fully understood, and its role in GC remains ambiguous [8]. Arginine, however, is more competitive in immune enhancement and is restricted to cancer cell progression at the metastatic stage [9]. However, the precise role of arginine in GC remains unclear [10].

The competitive mechanisms of arginine utilization during NO synthesis in cancers remain unclear. NO inhibition is still under debate, and dimethylation of arginine synthesis, while derivatives of arginine, i.e., ADMA and SDMA, and protein arginine methyltransferases (PRMTs) are actively involved in various cancers [11,12]. ADMA is usually metabolized to citrulline and dimethylamine (DMA) in the presence of dimethylarginine dimethyl aminohydrolases (DDAHs) [13] through numerous derivatives that are associated with inflammatory sites and dysregulated nitric oxides and DDAH1 activity in cancer patients. Studies have shown that arginine methylation of cellular proteins, which actively interact with the abnormal influx of ADMA in various cell types, such as endothelial and smooth muscle cells, is influenced by endogenous L-arginine, ADMA, and SDMA in gastric mucosal cells [14].

This growing evidence remains inadequate and unclear regarding the accumulation of ADMA and its bioavailability in GC cells/tissues. Mitochondrial dysfunction and calcium ionization irregularities have been linked in several cancers. Hence, intracellular calcium is needed to support an efficient repair or reinforcement of tight junctional proteins. Tight junctional (TJ) proteins, such as Claudin(s), ZO1, and Zonulin, are localized to the apical region, and their expression has prognostic value in some cancer patients [15]. Various TJ proteins (Claudin(s), ZO1, and Zonulin) are appropriately localized to the apical region, but the mechanisms underlying their remodeling remain unclear. Moreover, mitochondrial DNA copy number (mtDNA-CN) alterations have been reported in cancers and are being used to assess cancer progression [16]. In this study, we aimed to elucidate the detailed interlinkages among TJ proteins (Claudin 4, 7, 18), mtDNA-CN variations, NO synthesis, ADMA, SDMA, PD-L1, and matrix metalloproteinase-7 (MMP-7) mRNA, which could provide pathogenic insights and potential therapeutic targets for assessing the early stages of GC.

This paper is available as a preprint on Research Square under the title “Liaison of Dimethylated Arginine Between PD-L1 and Its Ligand Expressions in Gastric Cancer” (https://doi.org/10.21203/rs.3.rs-6800273/v1).

## Materials and methods

**Figure 1:**
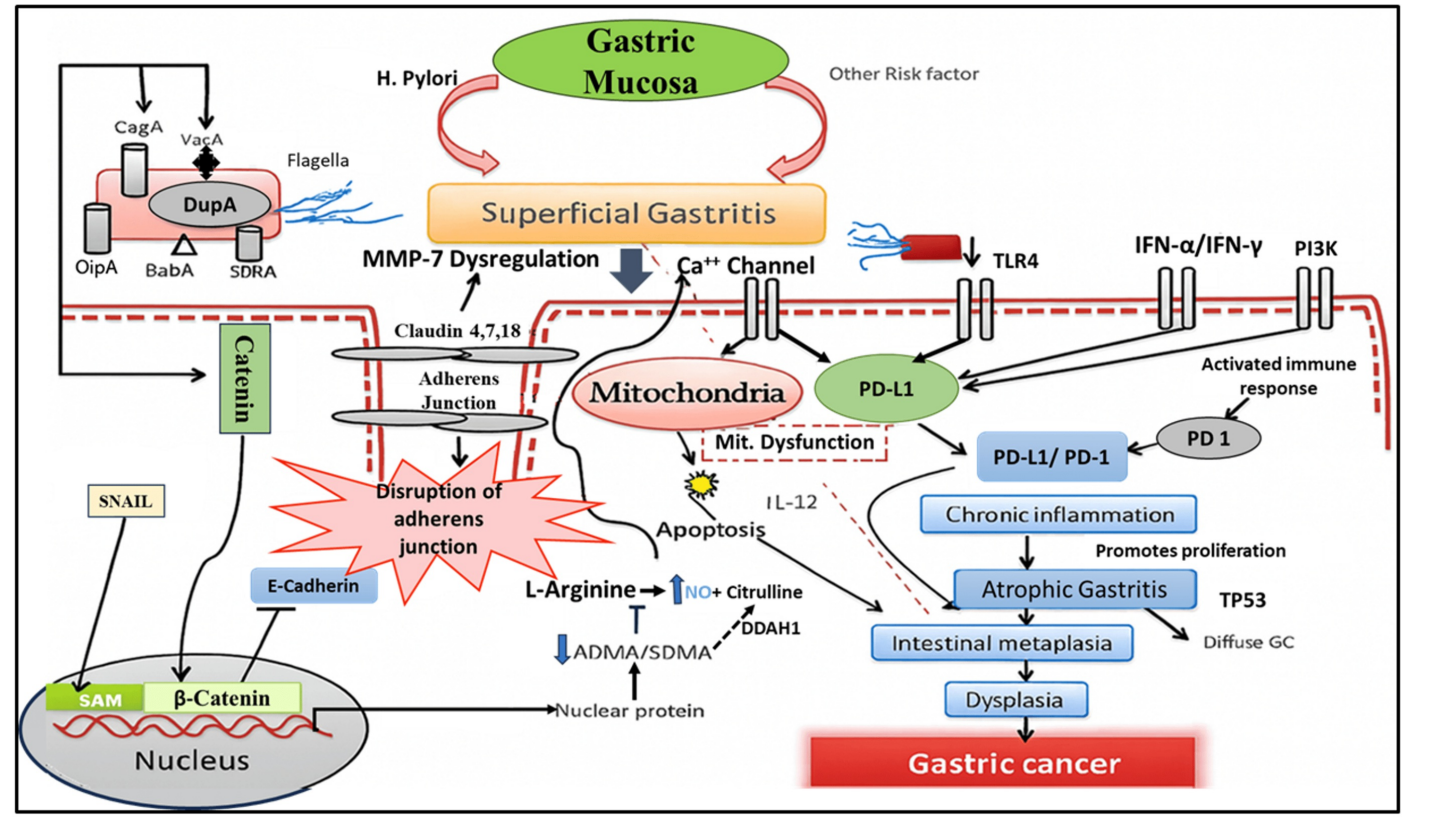
Pathomical pathway of gastric cancer. This figure was created using Biorender (https://www.biorender.com/). SAM: S-adenosylmethioninel PI3K: phosphoinositide 3 kinase; TLR-4: toll-like receptor-4; OiPA: outer-inflammatory protein A; CagA: cytotoxin-associated gene A; MMP-7: matrix metalloproteinase-7; PD-L1: programmed death-ligand 1; DDAH1: dimethylarginine dimethylaminohydrolase 1; IFN: interferon; IL: interleukin

The pathomical pathway of GC, with respect to PD-L1, MMP-7, and reactive oxygen species, is shown in Figure 1.

Patient recruitment

Patients were recruited from the Department of Gastroenterology and Dr. B.R. Ambedkar Institute Rotary Cancer Hospital, All India Institute of Medical Science, New Delhi, between 2016 and 2019. Eligible participants included individuals who underwent endoscopic evaluation for GC followed by total or subtotal gastrectomy. Blood and tissue samples were collected from the Outpatient Department (OPD) and endoscopy laboratory for molecular and biochemical testing and stored at -80°C until analysis. Histologic evaluation was performed according to Laurens’ classification of tumor grade and patient diagnosis [17]. Biopsies were collected from the targeted regions and from adjacent localized areas of the same patient. Stomach biopsies were also collected from disease control patients, such as those with dyspepsia. All procedures were performed as outlined in the protocol declaration. We performed histological examinations of all biopsies to categorize GC stages. Of these, 78% were moderately well differentiated, and 8% were designated as signet ring cell types. Overall, 36% of patients were diagnosed with low-grade cancer (grades I and II), and 64% of patients were diagnosed with high-grade cancer (grades III and IV). The age range of all recruited patients was 36 to 62 years.

Arginase activity

Arginase activity was measured at 570 nm in kinetic mode according to the manufacturer’s instructions using a photometric assay kit (ab180877; eLab Science, USA).

RNA extraction, cDNA synthesis, and quantitative polymerase chain reaction (q-PCR)

RNA was extracted from frozen specimens using TRIzolTM reagent (Thermo Fisher Scientific, Inc., Waltham, MA, USA) according to the manufacturer’s protocol. cDNA Synthesis using Superscript II reverse transcriptase kit (Thermo Fisher Scientific Real-time PCR (Agilent Technologies, CA, USA) was performed using SYBR chemistry with the following primer pairs, designed using Beacon Designer 5.1 Software (Premier Biosoft, Palo Alto, CA) for each target gene and synthesized by IDT, Canada: PD-L1 (F-5′-CCAAGGCGCAGATCAAAGAGA-3′; R-5′-AGGACCCAGACTAGCAGCA-3′), MMP-7 (F-5′-CATTTGATGGGCCAGGAAAAC-3′; R- 5′-GCAGCATACAGGAAGTTAATCC-3′), eNOS (F-5′-CGGCATCACCAGGAAGAAGA-3′; R-5′- CATGAGCGAGGCGGAGAT-3′), Claudin-4 (F-5′-AGCTCTGTGGCCTCAGGACTCT-3′; R-5′-CTCTTCTTAAATTACAA-3′), Claudin-7 (F-5′-ATGGCCAACTCGGGCCTGCAACTG-3′; R-5′-AGTGATGAATAGTC ACACGTATTCCTTGGAGGAATT-3′), Claudin-18 (F-5′CGGGCGGCCAGGATCATGTC-3′; R- 5′- ACTGCCTGCAGCATGGCTGG-3′), mtDNA (F- 5′-TGGCCATGGGTATGTTG TTA-3′;R-5′-TCTCTGCTCCCCACCTCTAAGT-3′), and glyceraldehyde 3-phosphate dehydrogenase (GAPDH) (F-5’-ACAGTCAGCCGCAT CTTC -3′; R-5’-GCCCAATACGACCAAATC-3′).

PD-L1, MMP-7, eNOS, Claudin 4, 7, and 18, and GAPDH mRNA expression

The 20 µL reaction mixture was prepared using 4 µL of cDNA, 10 µL of 2X SYBR® PCR Master Mix (Promega, USA), 1 µL of forward primer (10 pM), and 1 µL of reverse primer (10 pM) each. The variable thermal condition for q-PCR was performed at various annealing temperatures for the expression of PD-L1, MMP-7, eNOS, Claudin 4, 7, and 18, as well as GAPDH mRNA. The reaction was performed using a q-PCR system (Agilent AriaMx, US) to amplify the target mRNA.

mtDNA copy number

DNA was extracted using the salt extraction method [18] for mtDNA copy number analysis by SYBR Green qPCR. β-Globin and nuclear DNA genes were used as an internal control for mtDNA copy numbers. The amplified product was quantified, and the results were reported as copy number (mtDNA) [19].

ADMA and SDMA assay

Plasma ADMA and SDMA were analyzed by high-performance liquid chromatography (Agilent, Infinity 1260) as described by Teerlink et al., with minor modifications to the method [20]. The sample derivatization was performed using o-phthalaldehyde and 9-fluorenylmethyl chloroformate, with a flow rate of 1.2 mL/minute in gradient mode at an excitation wavelength of 254 nm and an emission cutoff filter of 324 nm.

DDAH-1 assay

The assay was performed according to the manufacturer’s instructions for the DDAH activity assay kit (Abcam, USA), measuring the absorbance in a microplate reader at 466 nm at room temperature. DDAH activity in the sample(s) was calculated using the following equations: DDAH (mU/mg) = (OD sample - ODSBC)/(OD (spiked sample) - OD sample) × 2 × T × C (nmol/minute × mg).

NO assay

Plasma total nitrite and nitrate levels were measured using the Griess reagent [21] at 540 nm with a spectrophotometer.

Statistical analysis

All statistical analyses were performed using RStudio (version 2024.12.1). Principal component analysis (PCA) was performed using the prcomp() function on z-scored data, and the results were visualized using ggplot2. Diagnostic accuracy was calculated using receiver operating characteristic (ROC) curves (pROC package), reporting area under the curve (AUC) with 95% confidence intervals (CIs), optimal Youden cutoffs, and Cohen’s d (effsize package). Inter-marker relationships were assessed using Pearson correlation and displayed as a heatmap. Group comparisons (GC, disease control (DC), healthy control (HC)) used appropriate parametric (t-test, analysis of variance) or non-parametric (Mann-Whitney U, Kruskal-Wallis) tests, following normality assessment (Shapiro-Wilk test). Hierarchical clustering (hclust ()) and final visualizations were generated using Complex Heatmap and ggplot2, respectively. A significance threshold of p-values ≤0.05 was applied to all statistical tests.

## Results

All patients were newly diagnosed with GC based on histopathological examination following endoscopic evaluation in the Department of Gastroenterology, All India Institute of Medical Sciences, New Delhi, India. The demographic profile of all groups, based on the inclusion and exclusion criteria for subjects, is categorized, and their clinical, biochemical, and haematological examinations are shown in Table 1. Adjacent tissues from the stomachs of GC patients were also collected by endoscopy, as were those from the disease controls, for histopathological examination.

**Table 1:** Demographic profile and baseline characteristics of study groups: hematological and biochemical profile. GC: gastric cancer; DC: disease control; HC: healthy control; MCV: mean corpuscular volume; MCHC: mean corpuscular hemoglobin concentration; ESR: erythrocyte sedimentation rate; AST: aspartate aminotransferase; ALT: alanine aminotransferase; ALP: alkaline phosphatase; MMP-7: matrix metalloproteinase-7; DDAH1: dimethylarginine dimethylaminohydrolase 1

Clinical parameters	GC (n = 25), mean ± SD	DC (n = 30), mean ± SD	HC (n = 20), mean ± SD	P-values
Demographic profile				
Age (years)	56.2 ± 8.8	45.9 ± 10.8	31.3 ± 6.5	NA
Male	20 (75%)	18 (60%)	10 (50%)	NA
Female	5(25%)	12 (40%)	10 (50%)	NA
Diarrhoea	3 (15%)	2 (6.6%)	None	NA
Constipation	2 (10%)	6(20%)	2 (10%)	NA
Pain abdomen	20 (100%)	12 (40%)	None	NA
Nausea	4 (20%)	4 (13.3%)	None	NA
Joint pain	2 (10%)	9 (30%)	None	NA
Pallor	6 (30%)	7 (23.3%)	None	NA
Fatigue	7 (35%)	9 (30%)	None	NA
Heart burn	18 (90%)	16 (53.3%)	None	NA
Weight loss	8 (40%)	3 (10%)	None	NA
Hematological profile				
Haemoglobin (g/dL)	11.56 ± 1.66	12.5 ± 1.0	12.9 ± 2.1	0.007
Hematocrit (%)	33.15 ± 5.03	39 ± 3.2	40.5 ± 5.8	0.004
MCV (fL)	85.34 ± 5.45	84.7 ± 4.0	83.9 ± 9.6	0.667
MCHC (g/dL)	31.8 ± 1.37	33.4 ± 1.8	31.9 ± 1.6	0.773
Total leukocyte count (10^3^/mm^3^)	7.13 ± 1.27	6.8 ± 1.3	7.2 ± 2.1	0.276
Platelets (10^3^/mm^3^)	170 ± 79.13	189.1 ± 50.3	206.9 ± 60.6	0.002
ESR	26.5 ± 8.30	16.2 ± 5.1	15.1 ± 11	0.001
Biochemical profile				
Serum urea (mg%)	26.27 ± 8.19	24.7 ± 3.9	22.7 ± 5.6	0.318
Creatinine (mg%)	0.82 ± 0.2	0.81 ± 0.1	0.74 ± 0.2	0.343
Calcium (mg%)	8.86 ± 0.49	8.9 ± 0.5	9.2 ± 0.3	0.047
Bilirubin (mg%)	0.6 ± 0.2	0.7 ± 0.2	0.7 ± 0.5	0.42
Total protein (gm%)	6.86 ± 0.55	7.8 ± 0.4	7.3 ± 0.3	0.00
ALT (IU/L)	26.36 ± 12.45	27.6 ± 5.9	27.3 ± 19	0.47
AST (IU/L)	24.81 ± 6.00	24.9 ± 8.1	25.3 ± 9.3	0.799
ALP (IU/L)	227.9 ± 84.98	232.7 ± 38.3	237.5 ± 37.1	0.767
Pathomic profile				
CgA (ng/mL)	1,008 ± 374.2	111.2 ± 42.8	28.7 ± 12.5	0.003
MMP-7 (pg/mL)	1,643.1 ± 2,113.9	131.7 ± 69.4	16.62 ± 8.62	0.001
Arginase activity (nM/minute/mg)	126 ± 44.62	42 ± 16.6	39.2 ± 21.84	0.0003
DDAH1 (pg/mL)	460 ± 218.2	188 ± 106	128.5 ± 96.6	0.0001
Nitric oxide (µg/mL)	19.2 ± 8.5	8.5 ± 1.4	7.1 ± 1.4	0.001

eNOS-mediated expression and NO dynamics in GC

**Figure 2:**
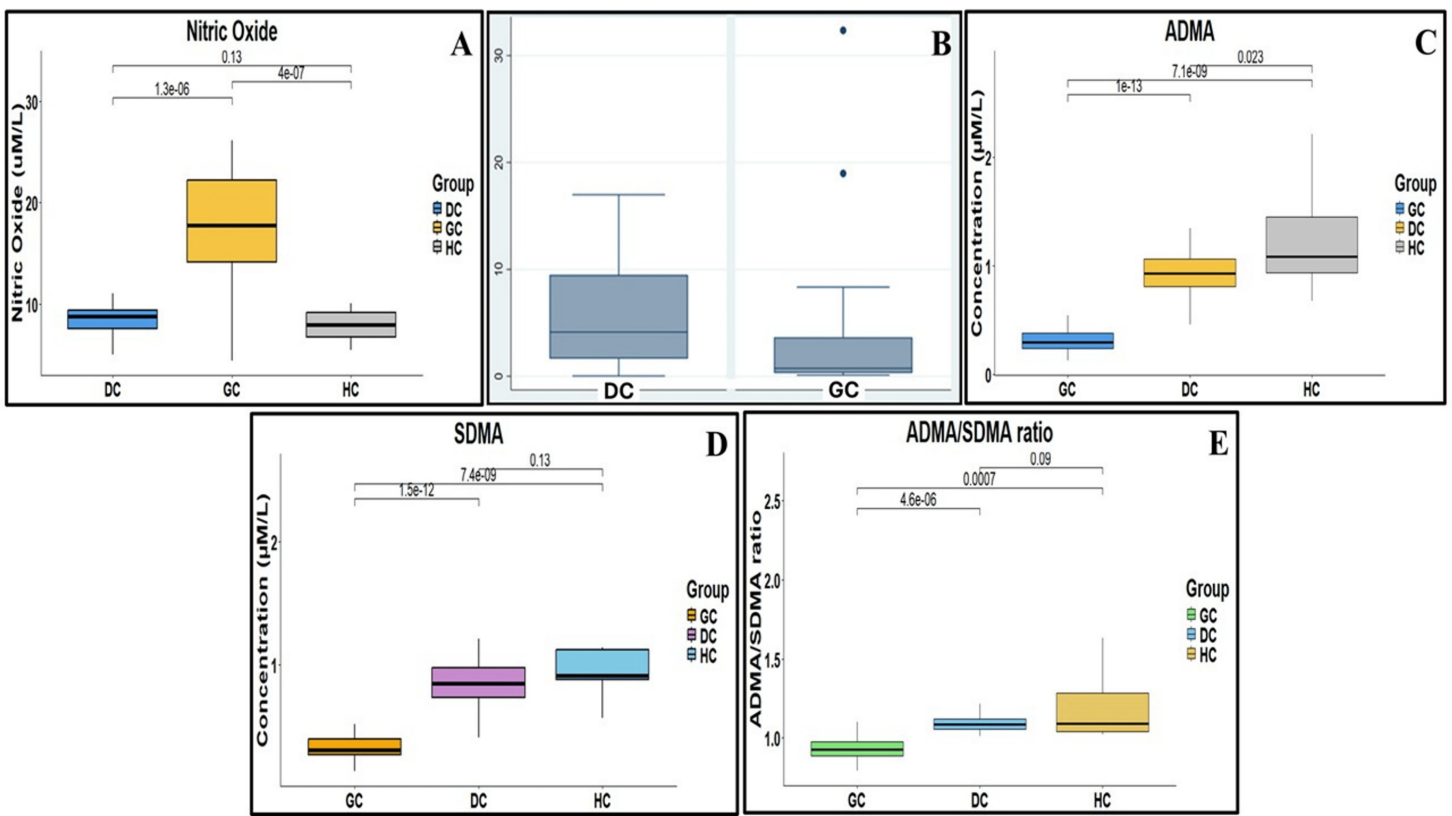
(A) Nitric oxide levels are represented by median lines, box ranges, and statistical comparison with p-values in GC, DC, and HC groups. (B) eNOS mRNA expressions in GC. (C) ADMA concentration. (D) SDMA concentration. (E) ADMA/SDMA ratio in GC, DC, and HC groups. Box-Whisker plots of different analytes were generated using RStudio (version 2024.12.1). GC: gastric cancer; DC: disease control; HC: healthy control; ADMA: asymmetric dimethylarginine; SDMA: symmetric dimethylarginine

NO levels were significantly higher, and the functional role of NO was examined by eNOS mRNA expression in 25 GC tissues and their adjacent gastric mucosal tissues. eNOS mRNA was significantly downregulated in GC patient tissues compared to disease controls and healthy subjects (Figures 2A, 2B). The ADMA and SDMA-mediated NO levels were compared between disease and healthy controls. The ADMA and SDMA levels were significantly reduced in GC (Figures 2C, 2D). GC patients exhibited the lowest arginine ratio, indicating impaired endothelial regulation and altered NO synthesis, with the ADMA/SDMA ratio being 21% lower than that of disease controls, as shown in Figure 2E.

MMP-7 levels and relative expression in GC

**Figure 3:**
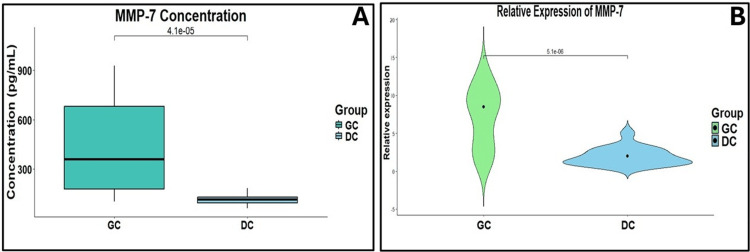
(A) Comparative distribution of circulating MMP-7 levels between GC and DC groups. (B) Relative expression of MMP-7 in GC and DC samples. Box-whisker and Violin plots were created using RStudio (version 2024.12.1). GC: gastric cancer; DC: disease control; MMP-7: matrix metalloproteinase-7

MMP-7 levels were also evidently higher in GC. The mean MMP-7 levels were 422.53 ± 280.25 pg/mL in GC versus 123.85 ± 55.37 pg/mL in disease controls (Figure 3A). At the mRNA level, relative expression was significantly higher in GC tissue, up fivefold compared to disease controls (Figure 3B).

PD-L1, Claudin-4, 7, and 18 mRNA expression in GC tissues and non-neoplastic mucosae

**Figure 4:**
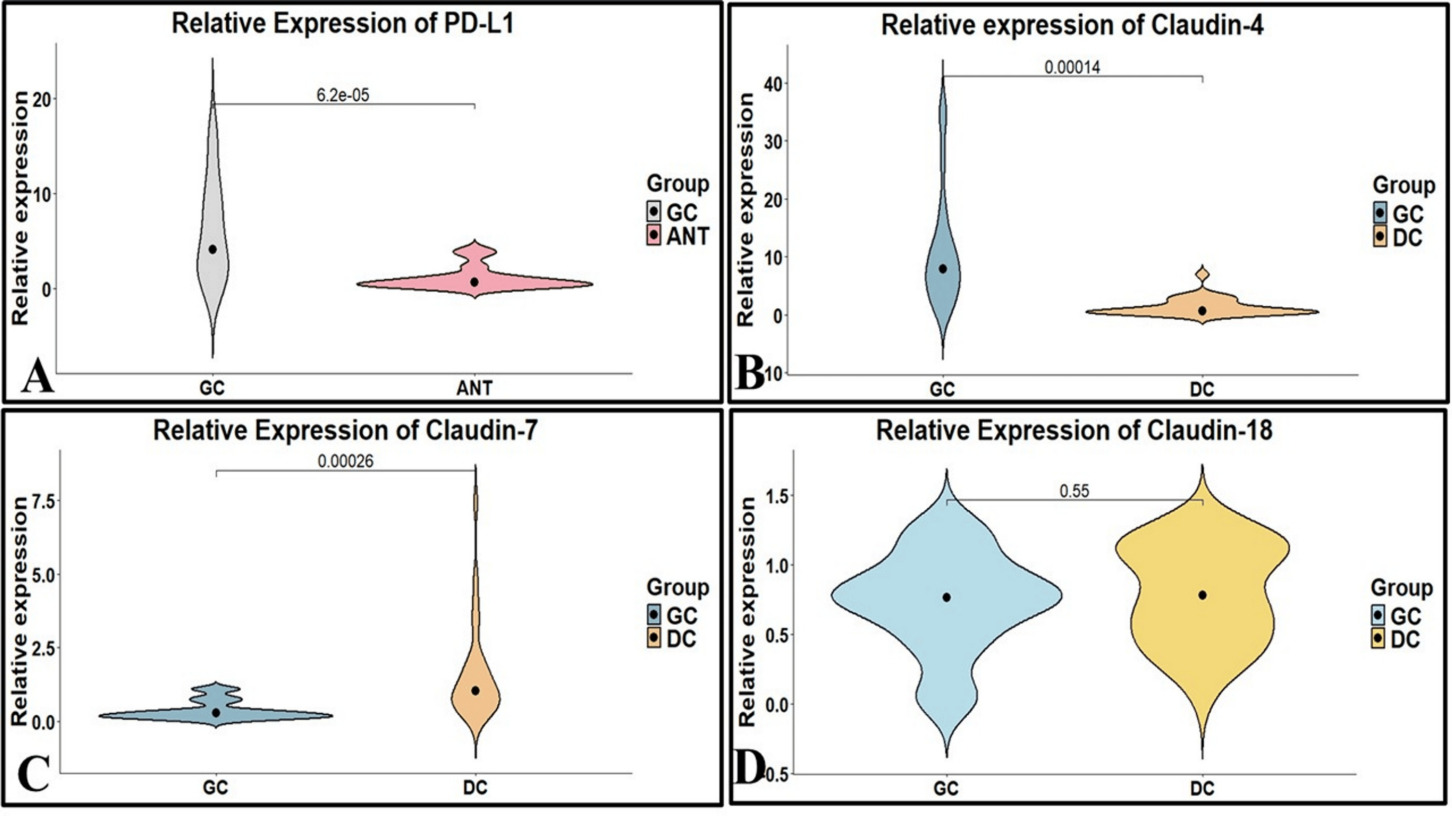
Relative expression of (A) PD-L1, (B) Claudin-4, (C) Claudin-7, and (D) Claudin-18 in GC and DC. The Violin plots were generated by using RStudio (version 2024.12.1). GC: gastric cancer; DC: disease control

PD-L1 was highly expressed in the Grade III and IV stages of GC patients than in lower grades of gastric cancer (p < 0.050, p < 0.010, respectively). The mRNA expression of PD-L1 was 6.20 ± 5.2 in GC, while the mean relative expression was 1.11 ± 1.22 in adjacent non-neoplastic tissue. A 3.6-fold overall change in PD-L1 expression was observed in GC patients (Figure 4A). The differential expression of Claudin-4 mRNA was overexpressed in GC compared to disease controls (Figure 4B). The differential expression of Claudin-7 mRNA was overexpressed in GC compared to disease controls (Figure 4C). However, a non-significant (p < 0.54) expression of Claudin-18 was found in GC compared with disease controls (Figure 4D), which supports a model of selective, rather than global, disruption of tight-junction components in GC.

Arginase enhances chromogranin A expression through DDAH1 activity in GC

Beyond altered arginase activity, Chromogranin A was significantly elevated in GC (p < 0.001). In contrast, DDAH1 activity increased in GC (Table 1), while the mtDNA copy numbers were lower, 60.40 ± 30.43 in GC and 80.57 ± 30.85 in disease controls (p < 0.05).

**Figure 5:**
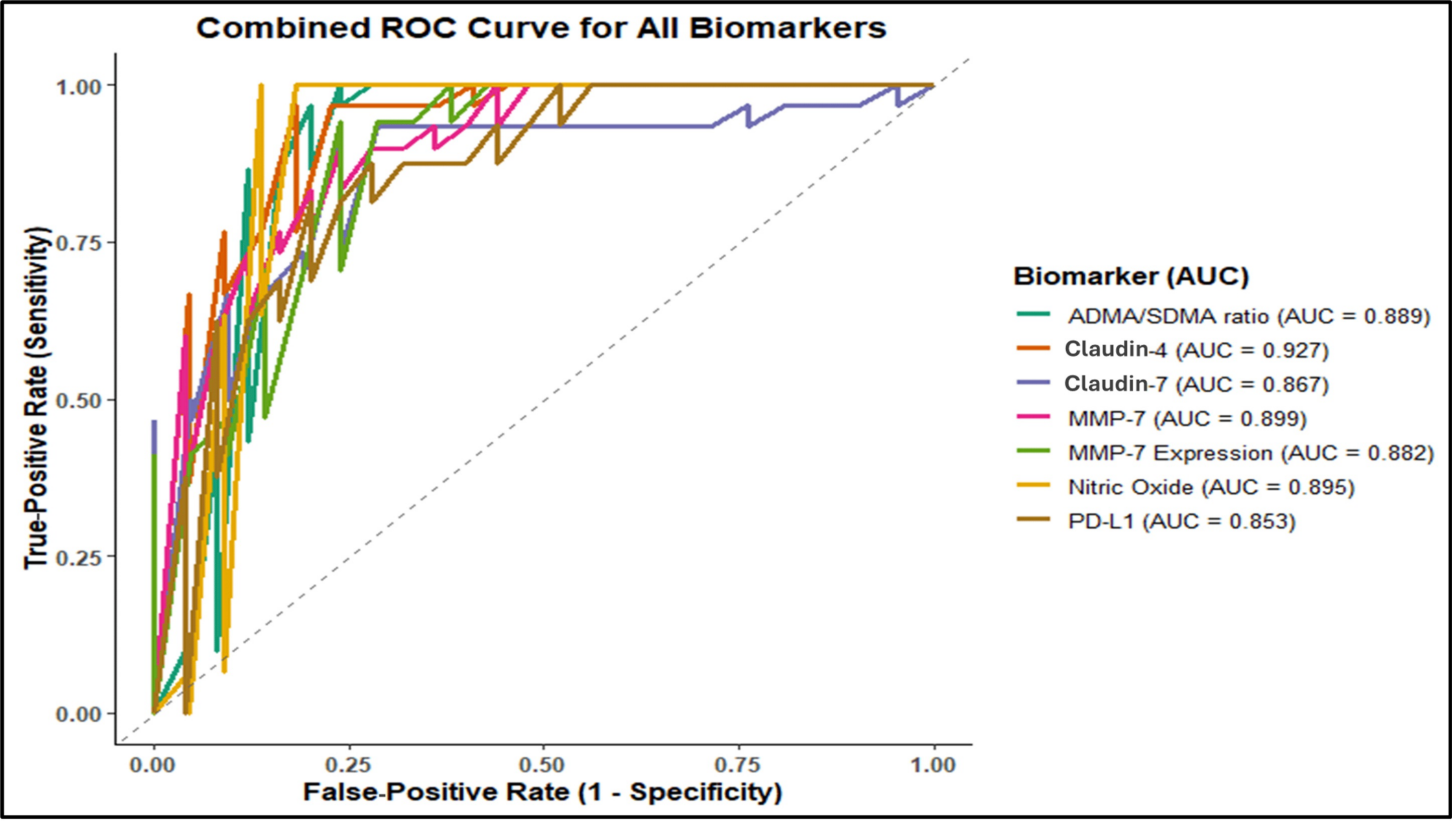
ROC curves of key parameters for distinguishing GC from DC. The ROC curve was generated using RStudio (version 2024.12.1). GC: gastric cancer; DC: disease control; ADMA: asymmetric dimethylarginine; SDMA: symmetric dimethylarginine; MMP-7: matrix metalloproteinase-7; PD-L1: programmed death-ligand 1; ROC: receiver operating characteristic; AUC: area under the curve

The resulting ROC curves revealed the potential of diagnostic accuracy, as shown in Figure 5, in terms of markers: Claudin-4 (AUC = 0.927), MMP-7 (AUC = 0.899), NO (AUC = 0.895), the ADMA/SDMA ratio (AUC = 0.889), Claudin-7 (AUC = 0.867), and PD-L1 (AUC = 0.853). This strong performance, with all AUC values ≥0.85, confirms their clinical utility and reflects the pronounced molecular alterations in GC.

Correlation of PD-L1 with other factors in GC

**Figure 6:**
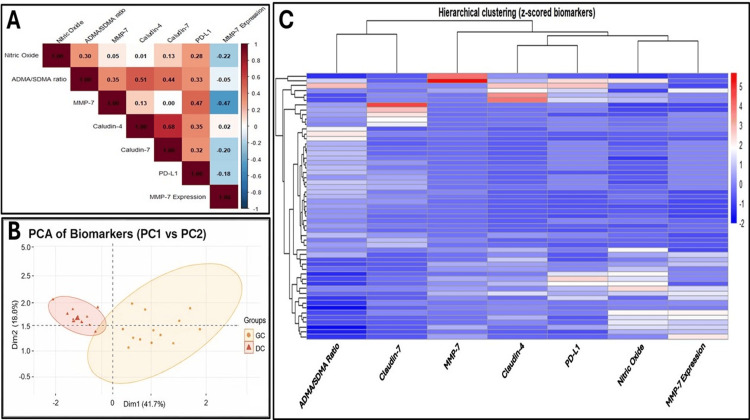
(A) Correlation matrix depicting positive correlations (red) and negative correlations (blue) among the parameters. (B) PCA scatter plot (PC1 vs. PC2). (C) Hierarchical clustering heatmap (z-scored biomarkers). These graphs were generated using RStudio (version 2024.12.1). GC: gastric cancer; DC: disease control; ADMA: asymmetric dimethylarginine; SDMA: symmetric dimethylarginine; MMP-7: matrix metalloproteinase-7; PD-L1: programmed death-ligand 1; PCA: principal component analysis

A comprehensive correlation matrix and an interconnecting molecular network linking immune evasion, metabolic dysregulation, and invasive processes were analyzed. The correlation matrix, depicted in Figure 6, shows (A) positive correlations (red) and negative correlations (blue) among the parameters, (B) PCA scatter plot (PC1 vs PC2), and (C) hierarchical clustering heatmap (z-scored Biomarkers). The invasion marker MMP-7 exhibits positive associations with both PD-L1 and NO, with its mRNA expression elevated over threefold in GC compared to disease controls. A strong positive correlation (r = 0.68) between Claudin-4 and Claudin-7 indicates coordinated epithelial remodelling, while a moderate correlation (r = 0.47) between MMP-7 and both Claudin-7 and PD-L1 suggests a mechanistic link between tissue invasion and immune evasion.

PCA shows a clear distinction, with PC1 (41.7% of variance) driven by epithelial and invasion markers (claudins, MMP-7) and PC2 (19.8%) capturing inflammatory mediators (NO, ADMA/SDMA). Overall, various statistical tools were applied to interpret the coordination effect among the groups, along with all measured analytics, to evaluate blood and tissue parameters using AUC, CI, Youden cutoffs, sensitivity, specificity, and Cohen’s d. Key performers include the ADMA/SDMA ratio (AUC CI = 0.766-0.987; Youden cutoff = ~1.028; Cohen’s *d* ≈ 1.54) and Claudin-7 (CI = 0.749-0.959; cutoff = ~0.384; *d* ≈ 0.987), demonstrating high diagnostic accuracy, stable precision, meaningful thresholds, and large effect sizes. In contrast, markers such as PD-L1 and MMP-7 exhibited low AUCs, wide or low CIs, and negligible effect sizes, indicating poor discriminatory ability. These metrics collectively validate the ADMA/SDMA ratio and Claudin-7 as robust, reproducible discriminators between GC and disease controls.

## Discussion

GC represents one of the most common malignancies worldwide, ranking fourth after lung cancer, breast cancer, and colorectal cancer. Even so, it remains the second most common cause of death. It is attributed to changes in lifestyle, higher tobacco consumption or smoking, alcohol intake, and increased stress levels in the population [22]. Our study reveals that PD-L1 expression was more pronounced in higher-grade tumors, specifically those above Grade III, than in lower-grade tumors. Elevated PD-L1 levels indicated increased tumor-associated immune evasion. The inhibitory responses rely on binding surface T lymphocytes and inducing T cells to undergo apoptosis. In this way, tumor cells can upregulate PD-L1 expression by activating inhibitory signals. Similar observations on PD-L1 expressions have already been reported in various cancers, including breast, ovarian, pancreatic, esophageal, colorectal, and gastric cancer [23,24].

MMP-7 mRNA expression was associated with GC severity by more than twofold, whereas other studies have shown no association in GC [25]. An interaction analysis between MMP-7 mRNA and the ADMA/SDMA ratio revealed a similar view of GC progression, as observed in this study, with a strong association with disease severity and higher GC tumor grades. These associations suggest that interactions among ADMA regulation, metabolism, export, and import could be a critical determinant of intracellular ADMA levels, the NOS substrate, and L-arginine availability. Increased NO production alters the relative oxidant-antioxidant homeostasis in GC. As NO has a dual role in disease progression at higher concentrations for long durations and releases peroxynitrite, which directly or indirectly damages DNA, leading to mutations and progression in GC.

DDAH1 accelerates the GC malignant process by upregulating the PD-L1 expression

An increased level of DDAH1 suppresses intracellular ADMA by activating eNOS expression, which simultaneously accelerates PD-L1 activity at inflammatory sites [26]. GC frequently upregulates PD-L1 on tumor and immune cells, engaging PD-1 on tumor-infiltrating CD8⁺ T cells to attenuate their proliferation, cytokine production, and cytotoxicity. High PD-L1 expression was observed and correlated with increasing tumor severity in GC patients, particularly Grade III. A substantial decrease in ADMA levels in GC patients and increased NO levels was noted; however, the exact mechanism underlying this progression remains under investigation [26]. In multiple tumor types, dysregulated DDAH/ADMA/NO signalling is associated with enhanced angiogenesis, vasculogenic mimicry, and tumor growth, with DDAH1 emerging as a key determinant of intratumoral NO availability and endothelial neovascularization responses [27].​ Methylated arginine derivatives, ADMA and SDMA, generated by PRMTs, competitively inhibit NOS; DDAH1 then degrades ADMA, so reduced ADMA together with increased arginase activity and preserved NOS can yield excess NO, polyamine production, and a pro-tumor “nitrergic” milieu, consistent with the observation of high NO and low ADMA in GC patients.

Synergy in the tumor microenvironment

The notion that PD-1 itself is “dimethylated into nitric oxide” is not appropriate; instead, PD-1/PD-L1 signalling and L-arginine/ADMA/NO metabolism are parallel axes that intersect at the level of T-cell function and cytokine-driven PD-L1 induction [28]. GC appears to couple disordered L-arginine/dimethylarginine metabolism with tight‑junction remodelling and immune checkpoint engagement. The integrated pattern described (low ADMA, high NO, arginase upregulation, high PD-L1, Claudin-4, and MMP-7, and reduced mtDNA copy number) is biologically coherent and potentially useful for biomarker development. However, mechanistic links between all elements are not yet fully resolved.​ Elevated Ca²⁺ activates transcription factors that drive MMP-7 mRNA and PD-L1 mRNA expression in GC.

Claudin‑4, MMP‑7, and junctional disruption

Claudins are the backbone of tight junctions and play a crucial role in regulating barrier function and the permeability of small molecules and ions. The expression of Claudin 4, 7, and 18 was altered in GC and disease control patients. Our finding that Claudin-4 and MMP-7 overexpression track with high PD-L1 and aberrant dimethylarginine/NO signalling aligns with an invasion-prone, inflamed microenvironment in which junctional proteins, MMPs, and immune checkpoints are co-regulated by shared upstream pathways (e.g., cytokines, growth factors, oxidative/nitrative stress). The loss of the tight junction structure, caused by aberrant expression of claudin proteins, suggests that a diffusion-based, nutrient-absorption process, along with other associated factors, may be involved in the survival and proliferation of cancer cells.

mtDNA copy number and nitrergic stress

A reduced mtDNA copy number in GC has been linked to mitochondrial genomic instability, altered oxidative phosphorylation, and adverse clinicopathological features. Even CgA is a stress-responsive protein and is associated with PD-L1, suggesting a link between inflammation and the tumor microenvironment, which drives both neuroendocrine features and immune evasion. However, some studies also report complex relationships between peripheral mtDNA copy number and survival. Excess NO and peroxynitrite, derived from a dysregulated L-arginine/ADMA balance, can damage mtDNA and respiratory complexes, providing a plausible mechanistic link from the “nitrigenic” pathway in GC.​

The ROC metrics reinforce and strengthen the findings in GC patients. Biomarkers exhibiting substantial biological differences, such as the ADMA/SDMA ratio and Claudin-7, also demonstrate high AUC values, stable CIs, meaningful Youden cutoffs, and large effect sizes. This convergence across statistical domains suggests the diagnosis of GC patients. Furthermore, the combined ROC curve indicates that multiple biomarkers simultaneously achieve high classification accuracy, suggesting that a multivariate biomarker panel may be more effective than any single marker in clinical settings.

Implications for biomarker panels in GC

Low ADMA and SDMA equilibrium with high arginase activity and NO, elevated PD-L1 (but with a continuum where suboptimal PD-L1 associates with better disease control), overexpression of Claudin-4 and MMP-7, and reduced mtDNA copy number matches emerging evidence that L-arginine metabolism, tight junction/ECM remodelling, immune checkpoint status, and mitochondrial dynamics jointly shape GC biology. As a working model, this constellation can be framed as an integrated biomarker axis of “nitrergic junctional immune mitochondrial” dysregulation in GC, testable in larger cohorts and potentially actionable via combinations of PD-1/PD-L1 blockade, arginase or DDAH-targeted interventions, and agents that stabilize mitochondrial function and epithelial barrier integrity.

ADMA/SDMA ratio and Claudin-4 mRNA expression were predominantly active at immune checkpoint sites, coordinating the progression of GC. Overall, the highlights of the study in GC are that the ADMA/SDMA ratio and Claudin-4 mRNA drive immune checkpoint progression, while MMP-7/PD-L1 mRNAs link to mtDNA decline and elevated ionized Ca²⁺. Although anti-PD-L1 therapies are being used in cancers and GC features, chromogranin A accumulation and excess arginine due to ADMA dysregulation are also observed. PD-L1 activity decompensation occurs through the interaction of reactive nitrogen species with the intra-entero lining of stomach cell walls, which may disrupt the microenvironment in tissues/adjacent cells by altering Ca²⁺ permeability and subsequently decreasing mitochondrial DNA copy numbers in GC. Therefore, the future biomarkers are ADMA/PD-L1 for progression, ADMA/SDMA-mtDNA for dimethylation severity, and DDAH1 inactivation, which disrupts NO/Ca²⁺ signalling to promote invasion. The study’s novelty shows that NO/MMP-7 overexpression impairs Ca²⁺ channels; reactive nitrogen species damage mitochondria, reducing mtDNA; low ADMA inhibits DDAH1; reactive nitrogen species-PD-L1 interactions alter gastric lining permeability, associating CgA/ADMA/SDMA with microenvironment disruption.

Limitations

Insufficient information was available regarding the nature of intratumor heterogeneity and sampling issues during biopsy specimen collection. Most patients visited the OPD in the end-stage or chronic stage of GC, often during the initial diagnosis. While recruiting patients for the series, we considered only cases with at least two biopsy specimens from different lesions (average number of biopsies per lesion: 1-2) to avoid multiple biopsy procedures in GC patients. As per national and international guidelines, this number (biopsies/tissue pieces) was reduced for evaluation in GC/GEC. Moreover, it was challenging to incorporate small preinvasive lesions into routine patient care. Thus, PD-L1 intratumor heterogeneity should be investigated in future studies considering surgical series of GC/GEC.

## Conclusions

Overall, the experimental results present a coherent, interlinked biological framework in which GC dysregulation is widespread across inflammatory, proteolytic, epithelial, and immune pathways. Overall, disrupted arginine dimethylation, mitochondrial dysfunction, and PD-1/PD-L1 signalling underscore a complex interplay that disrupts vascular regulation, angiogenesis, and immune evasion mechanisms in GC. Claudin-4, MMP-7, and PD-L1 mRNA, along with ADMA and mtDNA levels, can provide a basis for developing novel diagnostic and therapeutic strategies for GC and highlight the value of integrated, multilevel molecular profiling in cancer differentiation studies.
